# Single-cell transcriptomics: an emerging tool in the study of cardiometabolic disease

**DOI:** 10.1186/s12967-014-0312-0

**Published:** 2014-11-06

**Authors:** Amit V Khera, Nehal N Mehta

**Affiliations:** Cardiology Division, Massachusetts General Hospital, 55 Fruit Street, Boston, MA 02114 USA; Section of Inflammation and Cardiometabolic Diseases, National Heart, Lung and Blood Institute, Bethesda, MD 20892 USA

**Keywords:** Transcriptomics, Single-cell, Next generation sequencing, Cardiometabolic diseases

The potential for single-cell transcriptomics, the systematic study of the cellular RNA component transcribed by RNA polymerase II, to facilitate research on cellular heterogeneity, disease-specific biomarkers, and networks of expression was recently highlighted in the *Journal of Translational Medicine* by Zhu et al. [1]. We read this editorial with great interest and believe it has important implications for future research into cardiovascular diseases.

As discussed by Zhu and colleagues, gene expression reflects carefully regulated interactions between genomic DNA, epigenetic modifications, and RNA post-transcriptional modifications. Careful study of the “trancriptome” has led to fundamental discoveries in characterizing temporal and spatial patterns of gene expression. Recent advances in both array- and sequence-based technologies are likely to accelerate application to cardiovascular research. For example, gene-expression profiling of peripheral-blood specimens has been studied as a means of avoiding invasive endomyocardial biopsy in surveillance for rejection after cardiac transplantation or cardiac catheterization in diagnosing obstructive coronary artery disease [2,3].

However, these initial forays have involved samples of thousands of cells with expression output reflecting an average across this population. Recent advances in technology and bioinformatics have enabled documentation of substantial heterogeneity across seemingly similar cells and enabled high-resolution transcriptomics of single-cells [4]. Although techniques are rapidly evolving to overcome existing limitations, single-cell transcriptomics studies generally involve: 1) Isolation of individual cells and subsequent lysis; 2) Reverse transcription of RNA into cDNA; 3) Amplication of cDNA library; 4) Analysis via microarrays or deep sequencing; 5) Bioinformatics processing to allow for quantification and comparison to reference libraries [5]. Ongoing research will facilitate improved cellular isolation, more comprehensive capture of both coding and noncoding RNAs, and distinguishing random or stochastic variation from true signals.

Single-cell genome and transcriptome analysis has clear utility for circumstances in which the cell population of interest is rare. For example, genetic testing of preimplantation embryos has become a routine aspect of *in vitro* fertilization for couples at increased risk of passing on heritable disorders [6]. Traditional techniques have involved biopsy of one or two cells from a preimplantation embryo and subjecting them to locus- and often family-specific analysis using either PCR or fluorescent *in situ* hybridization assays. However, ongoing work has sought to generalize this process using array-based single nucleotide polymorphism assessment, comparative genomic hybridization that allows for chromosomal aberration assessment and next-generation sequencing that allows for detection of *de novo* mutations in an unbiased fashion [7]. Transcriptome profiling would allow for systematic assessment of the perturbations-induced by these abnormalities and further assist in preimplantation counseling and selection. Similarly, tumor cells circulating in peripheral blood can be isolated for noninvasive studies of human malignancy [8]. For example, microfluidic-based isolation and expression profiling of individual breast cancer cells from humans demonstrated marked variability in expression of cancer-related genes that would have been obscured if a larger population of cells was analyzed simultaneously [9].

Moving forward, researchers will increasingly be able to utilize single-cell expression profiling tools in the study of cardiovascular disease. For example, intravenous injection of low-dose LPS to induce experimental endotoxemia is a well-validated method of studying cardiometabolic phenotypes including adipose inflammation and insulin resistance [10]. RT-PCR assessments in adipose biopsies note dramatic increases in mRNA levels of the inflammatory cytokines interleukin-6 and TNF-alpha, among others. However, single-cell RNA sequencing of bone-marrow-derived dendritic cells after exposure to LPS noted a bimodal distribution of both expression patterns and RNA splicing across populations of seemingly similar cells [11]. The increased resolution of these analyses may improve future LPS-challenge experiments by allowing for the identification of relevant subtypes of cells, increase understanding of cellular interactions, and provide the foundation for regulatory network assembly.

Exponential decreases in the cost of sequencing have led to an explosion discovery of genetic links to cardiovascular disease over the last 10 years, driven largely by genome-wide association studies (GWAS) that allows for efficient phenotyping and unbiased analysis for association with a trait of interest. However, deciphering the causal and mechanistic pathways underlying these associations has proven challenging. For example, a 2012 review outlined 33 loci associated with myocardial infarction or coronary artery disease, of which the mechanism was unknown for 22 [12]. Alterations in gene expression are likely to play a key role in a number of these associations. For example, a common polymorphism at the 1p13 locus linked to both LDL-cholesterol and myocardial infarction creates a transcription factor binding site that modulates hepatocyte *SORT1* expression [13]. The 9p21 locus linked to atherosclerosis and myocardial infarction is thought to mediate its effect by inducing expression of ANRIL, a noncoding RNA, involved in regulation of the cell cycle [14].

Despite some success stories, it has become clear that thoughtful investigation of genotype-phenotype correlations will be needed to further understand complex cardiovascular diseases. Some of these will occur at the organismal or tissue level. For example, carriers of the R19X variant at the *APOC3* gene, involved in breakdown of circulating fats have substantially decreased postprandial serum triglycerides when assessed after an experimental high-fat challenge [15]. However, integration of genetic, epigenetic, transcriptomic, and proteomic technologies in data from single cells in tissues of interest are likely to emerge as powerful tools in the future. Quantifying allele-specific expression within a single cell is likely to provide important insights into gene regulation [16]. If tissue from a genotype of interest is unavailable, genome editing of pluripotent stem cells are likely to serve as viable surrogates [17]. These single-cell platforms can then serve as valuable reagents in studying the impact of pharmacologic, molecular, or environmental perturbations on the transcriptome.

We look forward to the coming era of “team science” in which investigators from the previously diverse fields of epidemiology, physiology, genomics, transcriptomics, and pharmacology come together in the development of novel biomarkers and pharmacologic therapies in the treatment of cardiovascular disease.
